# Whole Blood Interferon-Gamma Assay for Baseline Tuberculosis Screening among Japanese Healthcare Students

**DOI:** 10.1371/journal.pone.0000803

**Published:** 2007-08-29

**Authors:** Katsuyuki Hotta, Toshio Ogura, Kenji Nishii, Tsuyoshi Kodani, Masaru Onishi, Yukito Shimizu, Arihiko Kanehiro, Katsuyuki Kiura, Mitsune Tanimoto, Kazuo Tobe

**Affiliations:** 1 Health and Environmental Center, Okayama University, Okayama, Japan; 2 Department of Respiratory Medicine, Okayama Institute of Health and Prevention, Okayama, Japan; 3 Department of Respiratory Medicine, Okayama University Hospital, Okayama, Japan; McGill University, Canada

## Abstract

**Background:**

The whole blood interferon-gamma assay (QuantiFERON-TB-2G; QFT) has not been fully evaluated as a baseline tuberculosis screening test in Japanese healthcare students commencing clinical contact. The aim of this study was to compare the results from the QFT with those from the tuberculin skin test (TST) in a population deemed to be at a low risk for infection with *Mycobacterium tuberculosis*.

**Methodology/Principal Findings:**

Healthcare students recruited at Okayama University received both the TST and the QFT to assess the level of agreement between these two tests. The interleukin-10 levels before and after exposure to *M tuberculosis*-specific antigens (early-secreted antigenic target 6-kDa protein [ESAT-6] and culture filtrate protein 10 [CFP-10]) were also measured. Of the 536 healthcare students, most of whom had been vaccinated with bacillus-Calmette-Guérin (BCG), 207 (56%) were enrolled in this study. The agreement between the QFT and the TST results was poor, with positive result rates of 1.4% vs. 27.5%, respectively. A multivariate analysis also revealed that the induration diameter of the TST was not affected by the interferon-gamma concentration after exposure to either of the antigens but was influenced by the number of BCG needle scars (p = 0.046). The whole blood interleukin-10 assay revealed that after antigen exposure, the median increases in interleukin-10 concentration was higher in the subgroup with the small increase in interferon-gamma concentration than in the subgroup with the large increase in interferon-gamma concentration (0.3 vs. 0 pg/mL; p = 0.004).

**Conclusions/Significance:**

As a baseline screening test for low-risk Japanese healthcare students at their course entry, QFT yielded quite discordant results, compared with the TST, probably because of the low specificity of the TST results in the BCG-vaccinated population. We also found, for the first time, that the change in the interleukin-10 level after exposure to specific antigens was inversely associated with that in the interferon-gamma level in a low-risk population.

## Introduction

Tuberculosis continues to be a heavy burden on human health [1]. Especially, healthcare workers are at increased risk of infection because of occupational exposure to tuberculosis patients, although in Japan this risk is lower than in the low- and middle-income countries [2]–[4]. Indeed, female nurses have a risk for developing active tuberculosis that is two-fold higher than that of the general population in Japan (odds ratio of 2.3) [5]. One of the important component of tuberculosis infection control is the routine screening of healthcare workers for latent tuberculosis infection and the administration of chemoprophylaxis to those who test positive. The tuberculin skin test (TST) has been the only practical means for this purpose in the past century.

At Okayama University Hospital, tuberculosis patients are incidentally referred to the hospital before a definite diagnosis has been confirmed, even though the hospital does not have a tuberculosis-specific ward. Thus, not only healthcare workers, but also healthcare students studying at our hospital are at risk of tuberculosis exposure. As an infection control policy, a TST must be taken by all healthcare students and is optional for first-year postgraduate students prior to the start of clinical training, similar to the policies of many other medical universities [6]. The main purpose for performing a TST in this situation is to identify the baseline immune status of the healthcare students in case of future contact with tuberculosis patients, rather than screening for latent tuberculosis infection, because of the very low estimated prevalence of tuberculosis infection among young people in Japan [7].

Unfortunately, the TST has several major limitations; one of them is the possible occurrence of false-positive test results in individuals vaccinated with bacillus-Calmette-Guérin (BCG) [8]. Especially, after the initial vaccination at birth, the majority of the Japanese population has been repeatedly vaccinated with BCG until recently, during childhood if a negative TST result was obtained when attending primary and junior high school. Such repeated vaccinations can lead to a significant and persistent influence of BCG on TST results [9]. Therefore, this limitation is quite problematic in Japan, especially for the precise detection of subjects without tuberculosis infections. Recently, a whole blood interferon-gamma assay has been introduced for the diagnosis of latent tuberculosis infection and active tuberculosis; this assay is based on a specific elevation in the interferon-gamma concentration that occurs as T cells respond to early-secreted antigenic target 6-kDa protein (ESAT-6) and culture filtrate protein 10 (CFP-10), both of which are specifically expressed by *Mycobacterium tuberculosis* but not by BCG strains. Recent studies have shown that this blood assay has a moderate sensitivity for the detection of latent tuberculosis infection in the range of 70–80%, but is highly specific (over 95%) as compared with the TST even in BCG-vaccinated patients with varying risks of infection with *M tuberculosis* or active tuberculosis [8], [10]. Thus, for our healthcare students–most of who had been vaccinated with BCG, had not yet started bed-side training, and were at low risk for tuberculosis infection–a more accurate identification of their baseline immune status might be obtained using this blood test, rather than the TST. However, the usefulness of this assay as a baseline screening test for healthcare students at the time of their entry into clinical studies has not been fully evaluated in Japan. Here, we prospectively compared the accuracy of the whole blood interferon-gamma assay with that of the traditional TST in healthcare students thought to be at a low risk of infection with *M tuberculosis*.

Unlike Th1-mediated cytokines such as interferon-gamma that promote the killing of infected cells, regulatory cytokines including interleukin-10 have been implicated in the suppression of in vitro antigen-specific cellular immune responses in tuberculosis. Their down-regulatory mediators may be important players in controlling the excessive synthesis of pro-inflammatory cytokines and subsequent tissue damage [11]. However, few studies have reported how such Th2 mediators respond after stimulation with ESAT-6 and CFP-10 antigens, and all of these studies were conducted in mice and calves [12], [13], not in human subjects. Therefore, we also investigated the potential influence of these specific antigens on the interleukin-10 concentration as well as the interferon-gamma concentration, and assessed the association between changes in the concentrations of these two cytokines when exposed to the above two antigens.

## Methods

### Participants

All participants in this trial were prospectively recruited at Okayama University, Japan, between May and July, 2006. The subjects consisted of medical, nursing and dental students who were enrolled at the beginning of their clinical training, all of who were older than 18 years of age. First-year postgraduate students were also recruited. Subjects who received both whole blood assay and TST were considered to be eligible for this study. The protocol was approved by the ethics review committee of Okayama University. After providing written consent, the subjects were asked to complete a questionnaire about possible risk factors for exposure to *M tuberculosis*. The following data were collected: history of prior tuberculosis or exposure to a person with tuberculosis, and other tuberculosis risk factors like having an immunosuppressive condition (i.e., malignant disease or diabetes mellitus) or having taken immunosuppressive drugs in the 3 months prior to enrollment. Information regarding any clinical symptoms, previous TST results, BCG vaccination status (as reported by the prescribing physician and based on questions about past BCG vaccination and scar inspection), and demographic, clinical, and radiological data was also collected at the time of enrollment. Human immunodeficiency virus testing was not performed because of the low prevalence of positive human immunodeficiency virus expected in this young, educated population.

### Test methods—TST and whole blood interferon-gamma assay

For the TST, 0.1 mL of tuberculin purified protein derivative (PPD) (Nippon BCG Manufacturing, Tokyo, Japan; equivalent to about 3 TU of PPD-S) was injected intradermally into the volar aspect of the forearm and the transverse induration diameter was measured 48 hours later, as described previously [14]. A one-step TST was then performed.

The whole blood interferon-gamma assay was performed using the QuantiFERON-TB-2G (Nippon BCG Supply, Japan) as described elsewhere [14], principally one month after the TST. A heparinized blood sample was collected from each subject by venipuncture. After overnight incubation with saline (nil control), phytohemagglutinin (mitogen-positive control), and the *M tuberculosis*-specific antigens ESAT-6 or CFP-10, the concentration of interferon-gamma in the four plasma samples from each subject was determined using an ELISA. We interpreted the test results, after subtracting the value of the negative control well from the IU amount of interferon-gamma measured in the wells stimulated by the *M tuberculosis*-specific antigens as follows: positive if the interferon-gamma concentration in either of the antigen wells was ≥0.35 IU/mL; negative if the concentrations in both antigen wells were <0.35 IU/mL and the concentration in the positive control well was ≥0.5 IU/mL; and indeterminate if the concentration in both antigen wells was <0.35 IU/mL and the concentration in the positive control well was <0.5 IU/mL in the positive-control well. The technicians performing the whole blood interferon-gamma assay had already been trained by the manufacturers and were unaware of the subjects' tuberculosis status in this study.

### Test methods—Whole blood interleukin-10 assay

We measured the protein level of interleukin-10 in whole blood incubated overnight with antigens, using an ELISA kit (Biosource, Camarillo, California, USA). This assay also involved the same two steps; after the blood samples were separately incubated with CFP-10 and ESAT-6 and the nil and mitogen (phytohemagglutinin) controls for 18 hours at 37°C, the concentration of interleukin-10 in the four plasma samples from each subject was determined using an ELISA. The detection limit for interleukin-10 was <0.1 pg/mL. The results for ESAT-6 and CFP-10 were expressed as the detected concentration of interleukin-10 minus the concentration of interleukin-10 in the respective nil control plasma. This blood assay was also performed in a blind manner.

### Statistical methods

Agreement between the tests was quantified using the κ statistic. A logistic regression analysis for positive TST results (induration≥15mm) and for positive blood assay results was performed to adjust the potential confounding factors including age, prior tuberculin skin test, number of BCG scars, student category and increase in the interleukin-10 concentrations when exposed to either of the antigens. The Mann-Whitney U test was used for comparisons among groups. Because our goal was to recruit all healthcare students commencing clinical training, we did not determine a formal sample size. All p-values were two-sided, and significance was set at a p-value of ≤0.05.

## Results

### Demographics of the participants

**Figure 1:**
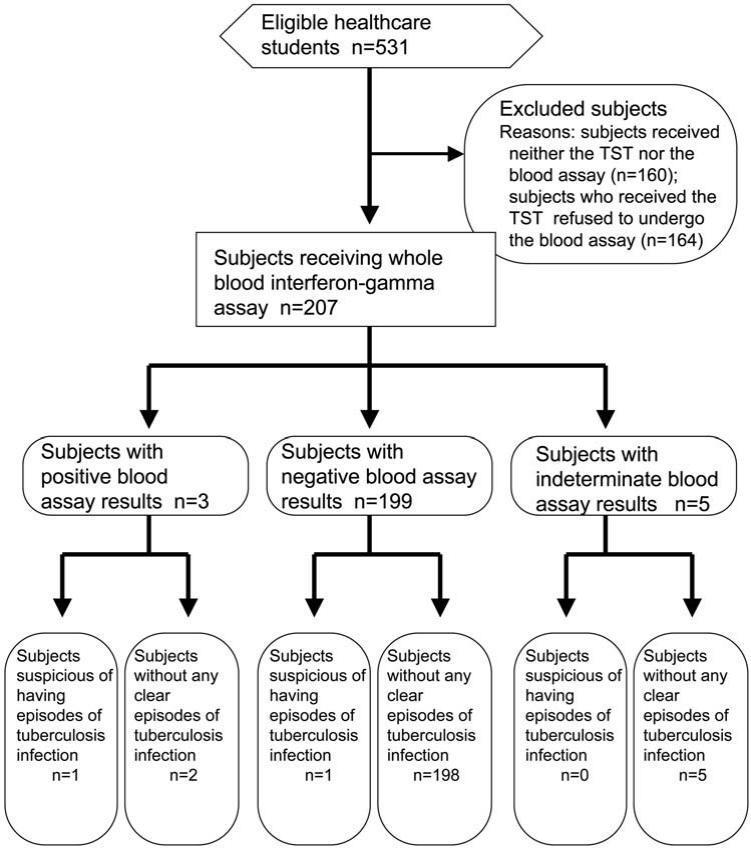
A flow diagram of the whole blood interferon-gamma assay in the 207 subjects.

Of the 536 students to whom the outline of this study was announced (Fig 1), 371 (69%) underwent the TST; of these students, 207 (56%) subjects also underwent the whole blood interferon-gamma assay, after providing their written informed consent. The demographics of the 207 participants are listed in Table 1. Only one of the 207 subjects had an obvious episode of close contact with a tuberculosis patient. This subject was a healthy 25-year-old nursing student who had been vaccinated with BCG soon after his birth. At the age of about six months, his grandfather, who had lived with him, contracted pulmonary tuberculosis and died. None of the family members who had been living with this patient have since developed tuberculosis, although their TST results at the time of the grandfather's death were not available. None of the subjects enrolled in this study reported having an immunosuppressive condition. The majority of the subjects had last been screened with the TST when entering junior high school.

**Table 1:** Demographics of 207 participants

Gender (male/female)	75 (36%)/132 (64%)
Median age (range)	20 (18–42)
Student groups	
Medical students	42 (20%)
Nursing students	107 (52%)
Dental students	44 (21%)
Postgraduate students	14 (7%)
Prior tuberculin skin test	
Yes/No	193 (93%)/14 (7%)
Number of prior BCG vaccinations	
0/1/2/3/4	3(1%)/91(44%)/79(38%)/10(5%)/10(5%)
Unknown	14 (7%)
Median age of subjects at which BCG was finally vaccinated (range)	
	7 (0–16)

Abbreviation: BCG = bacillus-Calmette-Guérin.

### Comparison of the TST and whole blood interferon-gamma assay results

**Figure 2:**
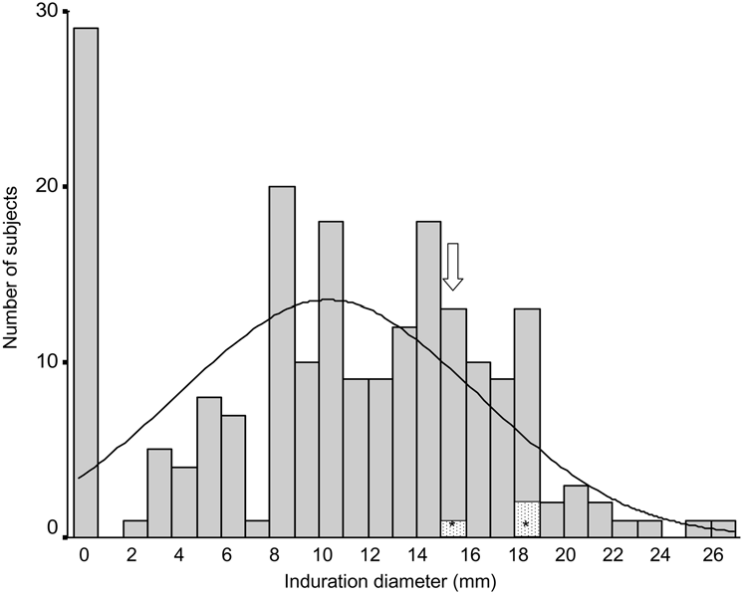
Distribution of tuberculin skin test and whole blood interferon-gamma assay results among 207 subjects. The bars for 15 mm and 18 mm included one and two subjects, respectively, with strong skin reactions (i.e., vesicles and hemorrhage) to the purified protein derivative (*). Three additional subjects with positive whole blood interferon-gamma assay results were included on the bar for 15 mm (arrow).

No treatments or interventions were undergone in the intervals between both tests. The two tests were safely conducted in all patients. Regarding the TST results, 171 subjects (82.6%) had an induration diameter of ≥5 mm, 124 (59.9%) had an induration diameter of ≥10 mm, and 57 (27.5%) had an induration diameter of ≥15 mm. The distribution of induration diameters is displayed in Fig 2. Three subjects (1.4%) had a strong reaction in addition to a large induration diameter (18 mm, 18 mm, and 15 mm), with obvious hemorrhaging in two subjects and vesicles in one subject. One of the three subjects had previously been in close contact with a tuberculosis patient, as mentioned above; since then, he had consistently exhibited a similar strong skin reaction whenever he received a TST, although his whole blood test was negative (ESAT-6: 0.03 IU/mL; CFP-10: 0.00 IU/mL). The other two subjects did not have relevant clinical symptoms, a history of close contact with a tuberculosis patient, or abnormal shadows on chest X-rays taken at the time of this study. These two subjects had received BCG vaccinations during childhood and had negative whole blood test results (ESAT-6: 0.00 IU/mL and 0.00 IU/mL; CFP-10: 0.03 IU/mL and 0.00 IU/mL).

Regarding the whole blood interferon-gamma assay (Fig 1), five subjects (2.4%) had indeterminate results because of insufficient interferon-gamma production in response to the mitogen. These indeterminate results were also used for the comparison of the two tests based on the entire sample data. The median baseline concentration of interferon-gamma (negative control) was 0.07IU/mL (range, 0.01 to 0.79 IU/mL), while the median increase in the interferon-gamma concentration after exposure to either of the antigens was 0.01 IU/mL (range, 0 to 1.89 IU/mL). Three subjects (1.4%) had positive blood test results (ESAT-6: 0.40 IU/mL, 0.37 IU/mL, and 1.89 IU/mL; CFP-10: 0.47 IU/mL, 1.26 IU/mL, and 0.03 IU/mL); these three subjects all had positive skin test results (induration of 15 mm each). None of these subjects had relevant clinical symptoms, a history of recent close contact with a tuberculosis patient, or abnormal shadows on chest X-rays taken at the time of this study. Two of the three subjects had been vaccinated with BCG during childhood, while the third had a positive TST test at birth and was thus considered to have had a tuberculosis infection despite not having had an obvious episode of exposure to a person with tuberculosis or a BCG vaccination.

Considering abovementioned two subjects would have previous episodes of tuberculosis infections (the first case with the strong TST reaction and the third case with the positive blood test result), the specificity of blood assay was calculated as 96.6% (198/205) (Fig 1). Regarding TST with a cutoff of induration diameter of ≥5, ≥10 and ≥15 mm, the specificity was 17.6%, 40.5% and 73.2%, respectively. In contrast, sensitivity was not assessed because of a few subjects with obvious tuberculosis disease in our study. The association between the diameter of the TST induration and the positive whole blood interferon-gamma assay result is shown in Table 2. Using cutoff levels of an induration diameter of ≥5, ≥10 and ≥15 mm for the TST and ≥0.35 IU/mL for the whole blood assay, the overall agreement between the two tests was 18.8%, 41.1% and 72.5% (κ = 0.007, 0.020 and 0.077), respectively. A logistic regression analysis also revealed that the diameter of the TST induration (≥15 mm *vs*. <15 mm) was not affected by the interferon-gamma concentration after exposure to either of the antigens (odd ratio = 1.441, 95% confidence interval = 0.737–2.818; p = 0.285), but was influenced by the number of needle scars from the BCG vaccination (odd ratio = 3.857; 95% confidence interval = 1.022–14.555; p = 0.046). In contrast, the whole blood assay result was not affected by the number of BCG scars (odd ratio = 0.905; 95% confidence interval = 0.207–3.958; p = 0.894).

**Table 2:** Agreement between whole blood interferon-gamma assay and tuberculosis skin test results

	TST Cutpoint, mm†		
<Results*>	**≥5**	**≥10**	**≥5**
Positive TST/positive INF-γ assay	3 (1.4%)	3 (1.4%)	3 (1.4%)
Negative TST/negative INF-γ assay	36 (17.4%)	82 (39.6%)	147 (71.0%)
Positive TST/negative INF-γ assay	163 (78.7%)	117 (56.5%)	52 (25.1%)
Negative TST/positive INF-γ assay	0	0	0
Any TST/indeterminate INF-γ assay	5 (2.4%)	5 (2.4%)	5 (2.4%)
Agreement, %	18.8	41.1	72.5
κ	0.007	0.02	0.077

†Longest transverse diameter of induration. Abbreviations: TST = tuberculin skin test, INF-γ = interferon-gamma. *IFN-γ assay cutpoint was at least 0.35 IU/mL.

### Whole blood interleukin-10 assay

**Figure 3:**
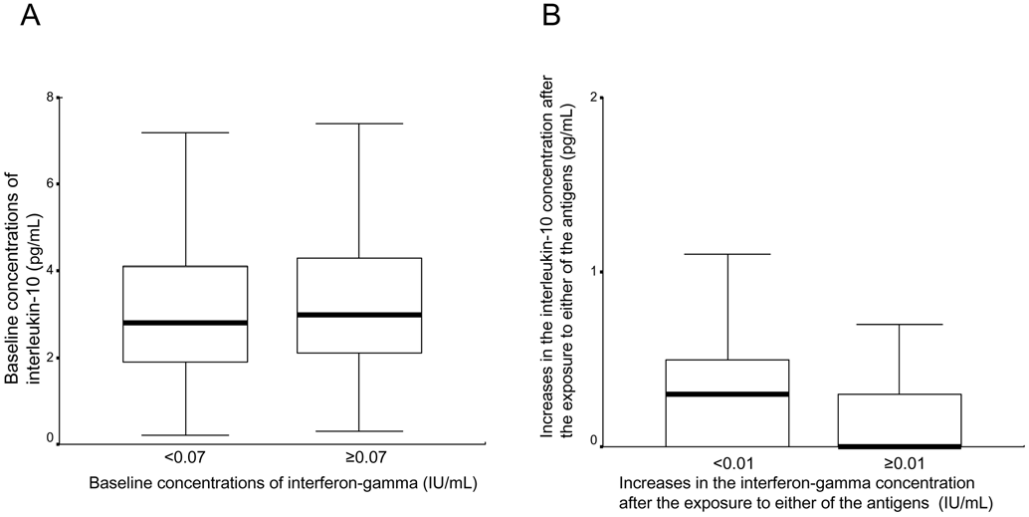
Interleukin-10 concentrations stratified by interferon-gamma concentrations. The median baseline concentration of interferon-gamma (negative control) was 0.07 IU/mL, while the median increase in the interferon-gamma concentration after exposure to either of the antigens was 0.01 IU/mL. A. Median baseline concentration of interleukin-10 (exposed to nil control) stratified by the baseline concentration of interferon-gamma (exposed to nil control) (2.8 vs. 3.0 pg/mL for the low [<0.07 IU/mL] and high [≥0.07 IU/mL] interferon-gamma concentrations; p = 0.263). B. Median increases in the interleukin-10 concentration stratified by increases in the interferon-gamma concentration after exposure to either of the antigens (0.3 vs. 0 pg/mL for the smaller [<0.01 IU/mL] and larger [≥0.01 IU/mL] increases in the interferon-gamma concentrations; p = 0.004).

This assay was assessable for all 207 subjects. The median baseline concentration of interleukin-10 (negative control) was 2.9 pg/mL (range: 0.2 to 39.9 pg/mL). This value was not associated with the baseline concentration of interferon-gamma (negative control) (median baseline interleukin-10 levels: 2.8 vs. 3.0 pg/mL for the low (<0.07 IU/mL) and high (≥0.07 IU/mL) baseline interferon-gamma concentrations, respectively; p = 0.263; Fig 3A). In contrast, when exposed to either of the antigens, the median increase in the interleukin-10 concentration was 0.1 pg/mL (range: 0 to 11.5 pg/mL) in the 207 subjects. When the median increase in the interferon-gamma concentration after exposure to the antigens (0.01 IU/mL) was applied as a cutoff value, the median increase in the interleukin-10 level was higher in the subgroup with the smaller increase in interferon-gamma concentration (<0.01 IU/mL) than in the subgroup with the larger increase in interferon-gamma concentration (≥ 0.01 IU/mL) (0.3 vs. 0 pg/mL; p = 0.004; Fig 3B).

## Discussion

Historically, infection control policies in many hospitals have recommended pre- and post-employment routine screening of healthcare workers using the TST [2]; in accordance with this recommendation, the TST has generally been required for healthcare students at the start of their clinical studies [6]. In Japan, however, repeated BCG vaccination has, until recently, been performed during childhood if a negative TST result was obtained while attending primary and junior high school. Healthcare students and healthcare workers with negative TST results at the time of their entry into clinical studies or at the time of hiring might also receive BCG vaccinations in Japan, despite no clear evidence of efficacy. Also, repeated testing using tuberculin during their employment may further boost their reaction to the TST. Thus, these factors may lead to a false-positive TST result [9], producing quite discordant positive results between the two tests both in a baseline screening setting for healthcare students (1.4% vs. 27.5% for the whole blood test vs. the TST; Table 2) and in a routine screening setting for Japanese healthcare workers who have been employed for one or more years (9.9% vs. 93.1%) [2].

To overcome the high false-positive TST results, several studies have been conducted to assess the specificity of interferon-gamma assays in low-risk healthcare students without active tuberculosis [14]–[16]. In an Australian study, the whole blood assay was performed in 60 medical students before and five months after BCG immunization [15]. Of note, the specificity of the TST decreased from 100% to 87% after BCG vaccination, while a high specificity (100%) was consistently observed for the whole blood assay, irrespective of BCG immunization. A previous Japanese study screened for tuberculosis infection in 213 nursing students deemed to be truly free of infection [14], a young population that was a quite similar cohort to ours. The study reported that 98% of the students were negative for the whole blood assay, while negative TST results (induration diameter≤10mm) were observed in only 35% of the same population. In our study, 96% and 40% of the subjects had negative blood assay and TST results, respectively (Table 2), suggesting that the results of both the blood assay and the TST are almost reproducible. Both studies also indicated that the blood assay rather than the TST corresponded to the estimated cumulative prevalence of tuberculosis infection in young people in Japan [7].

In contrast, Pai et al. recently showed concordant results between the two tests in Indian medical and nursing students at the start of their clinical studies [16]. Unlike the above-mentioned studies [14], [15]. and ours, this Indian study suggested that BCG vaccination had little impact on the TST results. Such minimal effect of the BCG vaccination might be more likely to occur when the vaccine is given to newborns only and in areas where the prevalence of tuberculosis is high. Considering the tuberculosis control policies of the Japanese government such as repeated BCG vaccination and the relatively low estimated prevalence of tuberculosis infection in Japan [7], the blood assay seems to be highly useful as a baseline tuberculosis screening program for our healthcare students, in whom the influence of BCG vaccination on the TST results is unavoidable. To confirm its usefulness, further comprehensive analyses are warranted, including a cost-effective study and a long-term follow-up study, especially in the three subjects who had positive blood assay results, the three subjects who had strong skin reactions to PPD but negative blood assay results, and the five subjects with indeterminate blood assay results. As a follow-up study, the Indian research evaluated serial whole blood interferon-gamma assays in medical and nursing students and found in their follow-up study that this assay showed promise for serial testing, despite the need to establish the optimal thresholds for distinguishing new infections from nonspecific variations [16]. Further research is also warranted to clarify the role of serial testing in countries with lower rates of tuberculosis incidence [17], [18].

One of our limitations is that we did not fully assess the sensitivity of the blood assay because of few subjects with active tuberculosis. The recent meta-analysis revealed the sensitivity of this assay did not seem to be significantly higher than that of TST despite its high specificity [9]. Indeed, one of our cases had a negative blood assay result despite the previous close contact with a tuberculosis patient and the strong TST reaction. Overall, the discordant results between the TST and blood assay we observed in this study might partly arise from its potentially low sensitivity of the blood assay in addition to the high false positive rate of the TST. These critical points should be further assessed in carefully designed population studies.

We should note another obvious limitation in our study-the possibility of bias due to a fairly high non-response rate. Only 207 (39%) of 536 students agreed to undergo both tests, and this may have introduced a selection bias that affected the positive TST and blood assay rates. Thus, our results should be carefully interpreted.

Interleukin-10 was first detected based on its cytokine synthesis inhibitory activity, mainly on macrophages [19]. The complementary activity of interleukin-10 and Th1-mediated cytokines, including interferon-gamma, may provide a mechanism for maintaining a balance between the protective immune response and excessive cellular activation. In this study, we also found evidence of this relationship; after exposure to either ESAT-6 or CFP-10, the change in the interleukin-10 concentration was inversely correlated with that of interferon-gamma (p = 0.004; Fig 3B), while no association between the baseline (nil control) concentrations of the two cytokines was seen (Fig 3A). Minion et al. reported comparable data demonstrating that mice splenocytes vaccinated with *Mycoplasma hyopneumoniae* surface antigen P71 alone produced higher levels of interleukin-10, while those vaccinated with an ESAT-6:P71 fusion protein secreted higher levels of interferon-gamma but failed to produce interleukin-10 [12]. Contrary to these results, Trajkovic et al. showed that CFP-10 could stimulate the secretion of interferon-gamma in mouse J774 cells in vitro, while CFP-10 pretreatment did not affect macrophage interleukin-10 synthesis [13]. Thus, the response of interleukin-10 to these specific antigens remains controversial in preclinical studies; however, our human data might encourage further investigation of whether interleukin-10 levels after exposure to specific antigens could be a useful tool for the detection of tuberculosis infection. Our limitation is that only one regulatory factor was assessed in relation to the interferon-gamma assay; there are indeed many cytokines related to homeostatic mechanisms balancing responses to specific tuberculosis antigens [20]. Thus, a more comprehensive study of profile of many other cytokines, rather than the assessment of a solitary member like IL-10 should be done in the future.

In conclusion, we found in this prospective study that the results of an interferon-gamma assay were quite discordant with those for the TST in a medical university setting, probably because of the influence of BCG vaccination on the TST results. Given this unavoidable influence of BCG vaccination and the relatively low estimated prevalence of tuberculosis infection in Japan, the blood assay seems to be useful as a baseline tuberculosis screening test for Japanese healthcare students, despite the need for further researches. Additionally, we report, for the first time, that the interleukin-10 level after exposure to specific antigens was inversely associated with the interferon-gamma level in the reported population.
